# JAK-STAT Signaling: A Double-Edged Sword of Immune Regulation and Cancer Progression

**DOI:** 10.3390/cancers11122002

**Published:** 2019-12-12

**Authors:** Katie L. Owen, Natasha K. Brockwell, Belinda S. Parker

**Affiliations:** 1 Cancer Immunology and Therapeutics Programs, Peter MacCallum Cancer Centre, VIC, Melbourne 3000, Australia; natasha.brockwell@petermac.org; 2 Sir Peter MacCallum Department of Oncology, University of Melbourne, VIC, Parkville 3052, Australia

**Keywords:** JAK, STAT, signaling, interferons, immunosurveillance, inflammation, cancer

## Abstract

Janus kinase-signal transducer and activator of transcription (JAK-STAT) signaling mediates almost all immune regulatory processes, including those that are involved in tumor cell recognition and tumor-driven immune escape. Antitumor immune responses are largely driven by STAT1 and STAT2 induction of type I and II interferons (IFNs) and the downstream programs IFNs potentiate. Conversely, STAT3 has been widely linked to cancer cell survival, immunosuppression, and sustained inflammation in the tumor microenvironment. The discovery of JAK-STAT cross-regulatory mechanisms, post-translational control, and non-canonical signal transduction has added a new level of complexity to JAK-STAT governance over tumor initiation and progression. Endeavors to better understand the vast effects of JAK-STAT signaling on antitumor immunity have unearthed a wide range of targets, including oncogenes, miRNAs, and other co-regulatory factors, which direct specific phenotypical outcomes subsequent to JAK-STAT stimulation. Yet, the rapidly expanding field of therapeutic developments aimed to resolve JAK-STAT aberrations commonly reported in a multitude of cancers has been marred by off-target effects. Here, we discuss JAK-STAT biology in the context of immunity and cancer, the consequences of pathway perturbations and current therapeutic interventions, to provide insight and consideration for future targeting innovations.

## 1. Introduction

Being identified as pivotal junctures of a multitude of pathways, signal transducer and activator of transcription (STAT) signaling mediates a vast array of processes that are required for homeostasis and development in mammals [1,2]. The myriad of cytokines and growths factors STAT activation induces drives events as varied as hematopoiesis, immune fitness, inflammation, tissue repair, adipogenesis, and apoptosis. In fact, the majority of immune responses initiated by cytokines are dependent on STATs. Unsurprisingly, perturbations in STAT signaling that result in either overactivation or global downregulation have been implicated in the emergence and progression of disease [3,4,5,6]. 

STAT activation and the cascade of downstream events is mechanistically simplistic when considered in isolation. Extracellular associations of numerous cytokines and other ligands with their respective transmembrane receptors lead to the activation of receptor-bound Janus kinases (JAKs). A succeeding conformational change that prevents inhibition by pseudokinase domains triggers the phosphorylation of resting STAT monomers in the cytoplasm, leading to homo- or heterodimerization and the formation of higher complexes, nuclear translocation, and DNA binding to specific palindromic sequences of target genes inducing their transcription and the further modulation of downstream targets [7]. These include many of the chemokines, caspases, interferon (IFN)-regulated genes (IRGs), growth factors, cyclin-dependent kinases, and metalloproteinases (MMPs) that are known to play a role in pathogenesis [3]. Yet, despite the seemingly linear order of events, the impact of mutations, selective dimerization, negative pathway regulation, and post-translational modifications of pathway members has branded the JAK-STAT axis a complex signaling cascade, of which many regulatory processes are still poorly understood. 

STATs have emerged as somewhat of a double-edged sword, being widely explored in the context of cancer. While several members of the STAT family, which encompasses STAT1, STAT2, STAT3, STAT4, STAT5A, STAT5B, and STAT6 as a whole, have been linked to tumor initiation and progression (STAT3 and STAT5), others are integral in antitumor defense and the maintenance of an effective and long-term immune response (STAT1 and STAT2) through evolutionarily conserved programs [1]. With increasing emphasis on immune-based agents as important therapeutics in the fight against solid tumor growth and spread, understanding the function of STAT specificity, redundancy, and connectedness in cancer is a critical component of achieving immunotherapeutic augmentation and success. 

Here, we investigate the good and the bad of STAT signaling in the context of immune regulation and cancer, and discuss how STATs can be targeted to bolster antitumor immune defense. 

## 2. JAK-STAT Signaling and Interferons

The presence of stimulatory or inhibitory signals governs the innate and adaptive immune activity that controls both effective immune surveillance and facilitates escape. Such signals determine the fate of plastic immune cells, such as T lymphocytes, and regulate their recruitment, survival, status, and eventual death [2]. Likewise, these signals may promote immune exclusion from the tumor microenvironment (TME), the expansion of immunosuppressive populations, such as myeloid-derived suppressor cells (MDSCs), unchecked tumor cell growth, and the desensitization of tumor cells to conventional therapies by promoting drug or radiation resistance [2]. Being comprised of various antigens, nucleic acids, foreign ligands, and the by-products of cellular decomposition, these signals also include cytokines and secreted factors that are produced following the induction of IFN, transforming growth factor-β (TGF-β), and nuclear factor kappaB (NFκB) pathways that are directly or indirectly regulated by JAK-STAT activation.

Interestingly, the JAK-STAT pathway was first identified within the framework of IFN signaling studies [8,9,10], including work exploring the processes regulated by the proinflammatory cytokine interleukin (IL)-6 [11,12,13]. Type I and II IFNs have both since been acknowledged as key mediators of antitumor immunity [14,15,16,17,18]. The former, including IFN-α and -β, has been linked to both transient and enduring immune activity against cancer cells by facilitating the priming of dendritic cells (DCs) required for T cell activation [19]; increasing tumor immunogenicity through the upregulation of major histocompatibility complex (MHC) molecules on antigen-presenting cells (APCs), which include tumor cells, to aid in specific errant cell recognition [20,21,22,23]; and, supporting immune cell migration, stimulation, and differentiation [16,24,25,26]. Furthermore, the binding of type I IFNs to their cognate IFN-α receptors induces canonical signaling pathways that regulate the PI3K/AKT/mTOR axis [27,28,29]. This, in addition to exerting a multitude of growth and survival effects, stimulates NFκB activity [30], which has now been evidenced to promote antigen presentation, the induction of IRGs, and the secretion of immune-activating cytokines [31,32,33,34,35,36] in addition to the known pro-tumorigenic role it plays in fueling chronic inflammation.

**Figure 1:**
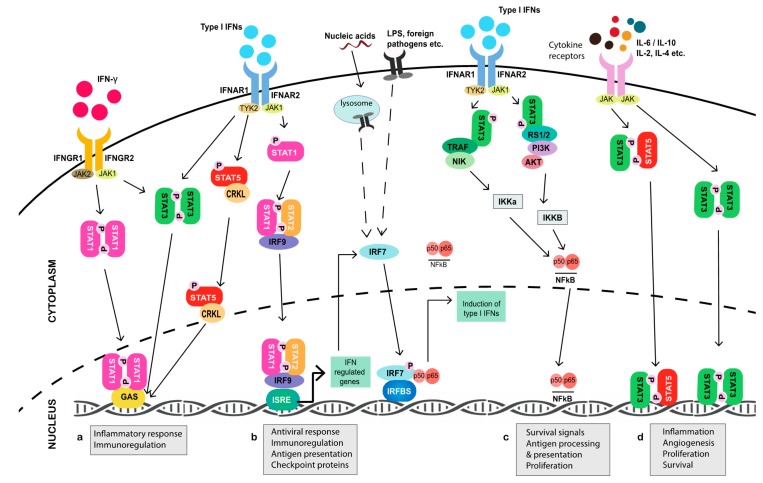
Janus kinase-signal transducer and activator of transcription (JAK-STAT) signaling pathways (simplified). Cytokine signaling occurs through various JAK-STATs. (**a**) Canonical type II IFN signaling occurs through receptors, IFNGR1 and IFNGR2, which constitutively associate with JAK1 and JAK2, respectively, leading to the phosphorylation of STAT1. Phosphorylated STAT1 homodimers translocate to the nucleus and bind to GAS elements, initiating the transcription of IFN-γ induced genes associated with immune activation. IFN-γ signaling can also lead to the phosphorylation of STAT3, which forms homo- or heterodimers that bind to GAS elements to induce inflammatory genes. (**b**) The type I IFN pathway can be stimulated by multiple family members, the most well-known being IFN-α and IFN-β. The receptors IFNAR1 and IFNAR2 are associated with TYK2 and JAK1, respectively. Canonical type I IFN signaling occurs through the phosphorylation of STAT1 and STAT2, which, together with IRF9, form the ISGF3 complex. ISGF3 translocates to the nucleus to initiate the transcription of IRGs through the ISRE regulatory sequence. Non-canonical type I IFN signaling can occur through the CRKL or NFκB pathway. Subsequent to JAK activation, CRKL can become phosphorylated by TYK2, which leads to CRKL complexation with STAT5, which then binds GAS elements in the nucleus. (**c**) IFNAR1/2 signaling through TYK2 and JAK1 can trigger the activation of the NFκB pathway through phosphoinositide 3-kinase (PI3K), protein kinase B (AKT), and TNF receptor-associated factors (TRAFs) that act through IKKa and IKKb to drive NFκB induction of genes associated with survival and cell proliferation. The production of type I IFNs can also occur through activation of PRRs that converge on IRF7 to promote further production of type I IFNs and viral response genes. (**d**) Cytokines, both pro- and anti-inflammatory, signal through their associated receptor/JAK complexes, resulting in the downstream phosphorylation of STATs (homo- or heterodimers). Translocation of these STAT complexes to the nucleus drives the transcription of genes involved in processes ranging from inflammation to angiogenesis and survival. *Abbreviations*: JAK, Janus kinase; STAT, signal transducer and activator of transcription; IFN, interferon; IFNGR, interferon gamma receptor; GAS, gamma activated sequence; IFNAR, interferon alpha receptor; ISGF3, interferon-stimulated gene factor 3; ISRE, interferon-stimulated response element; TYK2, tyrosine kinase 2; NFκB, nuclear factor kappaB; PI3K, phosphoinositide 3-kinase; AKT, protein kinase B; TRAF, TNF receptor-associated factor; PRR, pattern recognition receptor.

While the production of type I IFNs can occur via multiple, proximal pathways that eventually converge on IFN regulatory factor (IRF) 7 [37,38]—the principal transcriptional inducer of IFN-α/β signaling [39,40]—type I IFNs canonically signal through their binding of integral membrane subunits IFNAR1 and IFNAR2 expressed on all cells (Figure 1). Binding then initiates receptor dimerization, which results in the phosphorylation of their associated cytoplasmic JAK1 and tyrosine kinase 2 (TYK2) enzymes. This leads to the recruitment of STAT1 and STAT2 monomers via their conserved Src homology 2 (SH2) phosphotyrosine-binding domains, which are phosphorylated and subsequently dimerize to facilitate the rapid binding of IRF9 in the cytoplasm to form the interferon-stimulated gene factor 3 (ISGF3) complex [41,42,43]. Once ISGF3 translocates to the nucleus, up to several thousand genes that contain an IFN-stimulated response element (ISRE) in their promoter region are transcribed, including *Irf7* itself [44,45]. Recent studies have evidenced that the upregulation of latent cytoplasmic STAT1, STAT2, and IRF9, in response to prolonged type I IFN stimulation, can lead to the translocation of unphosphorylated (U)-ISGF3 to the nucleus to induce IRG expression, while the phosphorylation of ISGF3 was previously thought to be a prerequisite of complex activation [46,47]. Such events parallel the ability of accumulated IRF7 to homodimerize at high concentration in the absence of phosphorylation to stimulate the production of endogenous IFN-α/β [48]. In a non-canonical cascade, U-STAT2 has also been evidenced to complex with IRF9 in the absence of STAT1 and, in cooperation with NFκB, bind both ISRE and κB elements of *Il6* to promote errant downstream STAT3 activation [49]. 

Immune cells, on the other hand, predominantly produce type II IFN (IFN-γ), and it plays a more direct role in tumor cell killing. In addition to promoting senescence [50] and autophagy-mediated apoptosis [51] in tumor cells, IFN-γ triggers, enhances, and enables the cytotoxic and lytic function of activated CD8^+^ T cells and natural killer (NK) cells [52,53,54]. Additionally, IFN-γ has been suggested to polarize macrophages toward a classical M1 phenotype, which are associated with potent antimicrobial and direct tumoricidal function, along with the stimulation of Th1 cytotoxic T cells [54,55,56] and production of immunomodulatory molecules [57]. Like type I IFNs, IFN-γ signals through two transmembrane receptor subunits, designated IFNGR1 and IFNGR2 [58]. Extracellular engagement of IFN-γ activates receptor-associated JAKs, which results in the selective recruitment, SH2-facilitated docking, and phosphorylation of STAT1, which homodimerizes to form γ—activated factor (GAF) and translocates to the nucleus to bind the gamma interferon activation site (GAS) elements of IFN-γ target genes [59]. However, there are exceptions to that rule, such as the transcriptional activation of the metastasis suppressor intracellular adhesion molecule-1 (ICAM-1) that is involved in the inhibition of M2 macrophage polarization [60], which only occurs when STAT1 synergistically interacts with SP1 at the GC box of the ICAM-1 promoter [61]. Interestingly, the complexation of GAF also occurs in response to type I IFN JAK-STAT activation. However, ISGF3 largely outcompetes GAF for STAT1 binding due to the higher affinity of ISGF3 complex formation and, as such, it is observed at a much lower concentration [62]. Nevertheless, IFN-α has been consistently reported to selectively induce proinflammatory genes containing GAS in the absence of an ISRE [63,64,65,66,67]. Moreover, it has recently been evidenced that U-STAT1 persists in macrophages following pathogenic stimulation it and could induce immunomodulatory genes, in contrast to phosphorylated (p)-STAT1, which undergoes rapid degradation [68]. 

Cooperation between type I and II IFNs underpins both innate and adaptive antitumor immune responses in which the availability of latent cellular STAT1 facilitates crucial pathway crosstalk [30,69]. Yet, the activation of other STAT complexes, including STAT3, STAT4, and STAT5, in direct response to IFNs along with the paracrine and autocrine effects of the many cytokines (IL-10, IL-4, IL-2, IL-17, IL-6, IL-23) and factors (MMP9, vascular endothelial growth factor (VEGF), HIF-1α, c-MYC) produced during JAK-STAT transduction, ensures that the impact of STAT-mediated signaling in the TME is extensive and as destructive as it is favorable [2].

## 3. Tumor Eradication and Immune Surveillance

As the field of cancer immunotherapeutics continues to expand, so does our understanding of the importance of the immune system in restraining tumour growth and metastasis. Genomic instability, cellular stress, altered metabolism, and mechanistic disruptions in apoptotic and cycling pathways are known to fuel the evolution of malignant cells [70]. Yet, not all malignant cells manifest fully-fledged tumors and not all tumors metastasize. This is, in no small part, due to the activity of immune cells and the immune-regulatory factors produced by stromal cells in the TME that help to orchestrate effective antitumor immune surveillance—processes chiefly driven by JAK-STAT signaling. 

**Figure 2:**
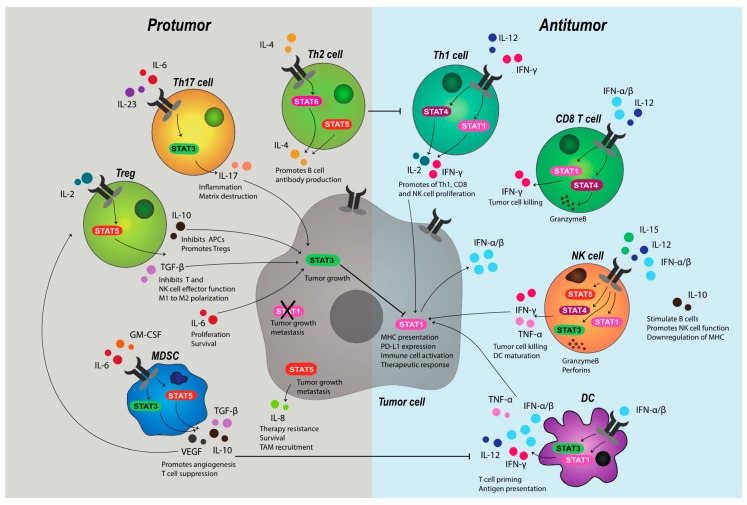
STAT signaling in the tumor microenvironment (TME). Tumor cell fate is governed by tumor-inherent properties and the activity of surrounding immune cells, both of which are influenced by JAK-STAT signaling. A protumor immune microenvironment reflects a permissive niche and is comprised of populations including Th2 and Th17 cells, Tregs, and myeloid-derived suppressor cells (MDSCs), which signal mainly through STAT3 and STAT5 to produce inflammatory cytokines, such as IL-1, IL-17, IL-10, TGF-β, or vascular endothelial growth factor (VEGF). These cytokines can inhibit antitumor immune responses, promote TME suppression and act directly on tumor cells. High tumor cell STAT3 promotes tumor growth and metastasis, inhibits STAT1 signal transduction and leads to the production of protumor chemokines, cytokines and growth factors. Antitumor immune signaling is largely facilitated by cells involved in antigen recognition and directed cancer cell killing. Th1 cells, CD8 T cells, natural killer (NK) cells, and dendritic cells (DCs) are all involved in antitumor immune responses through the secretion of cytokines, such as IFN-α/β, IFN-γ, TNF-α, and IL-2, along with perforins and granzymes, that induce tumor cell apoptosis, necrosis, T cell priming and antigen presentation. Cell-specific function and selective cytokine production is mainly regulated through STAT1 and STAT4. Acting on the tumor cell, these cytokines can signal through STAT1 to promote tumor immunogenicity via the upregulation of MHC and checkpoint proteins. Tumor cells themselves can also produce immune-stimulating cytokines, such as type I IFNs, to further promote immune infiltration and tumor visibility. *Abbreviations*: JAK, Janus kinase; STAT, signal transducer and activator of transcription; Th, T-helper; Treg, regulatory T cell; MDSCs, myeloid-derived suppressor cells; IL, interleukin; TGF-β, transforming growth factor-beta; VEGF, vascular endothelial growth factor; TME, tumor microenvironment; NK, natural killer; DC, dendritic cell; IFN, interferon; TNF-α, tumour necrosis factor-alpha; MHC, major histocompatibility complex; GM-CSF, granulocyte-macrophage colony stimulating factor; PD-L1, programmed death-ligand 1.

Early, non-specific responses to tumor cells are thought to be executed by natural killer (NK) cells. Indeed, improved clinical outcomes in several cancer types have been linked to high tumor expression of NK cell ligands, such as MIC-AB and ULBP-2 [71]. In mice, it has been widely evidenced that those deficient in NK cells are more susceptible to tumors [25,72,73]. Similarly, in humans, suppressed NK cell function and low infiltration has been observed in the TME of high-grade ovarian carcinoma [74], metastatic lung adenocarcinoma [75], and gastric cancer [76], respectively, while low NK cell-mediated cytotoxicity in pan-cancer patients correlates with increased incidence and risk of progression [77]. The development, maturation, activation, and function of NK cells is tightly regulated by cytokines that both induce and are induced by JAK-STAT pathways (Figure 2). In a convoluted process, IL-2 and IL-15 promote NK cell homeostasis, proliferation, and function [78,79,80], through STAT5 transduction, while IL-12 mediates NK cell activation and cytotoxicity exclusively, primarily via the activation of STAT1, STAT3, and STAT4 to produce IFN-γ, perforin and granzymes [26,81,82]. Competent IFN-α/β signaling, as modulated by STAT1-STAT2, has been shown to be integral for effective cytokine-mediated responses by NK cells, particularly in the acquisition of IL-2-driven cytotoxic function and IL-15 production and priming through cognitive receptor expression [25,83,84]. Intact STAT1 signaling has also been shown to protect T cells from NK cell killing [85], which might have implications in an immune suppressed TME in which downregulation of T cell MHC, and thus loss of ‘self’, can occur in response to alterations in IFN availability. Indeed, it is well established that MHC deficiency—widely reported in metastatic cancers and linked to T cell inefficacy—can trigger NK cell reactivity [86,87]. 

The activated NK cells also aide T cell responsiveness by promoting DC maturation and release of IL-12, which acts directly on both CD8 and CD4 T helper (Th) cells to enhance tumor clearance [88]. This, in addition to the myriad of biological consequences JAK-STAT signaling manifests in the adaptive arm of immunity, has cemented JAK-STAT transduction as a critical component of tumor cell killing and specific, long-term memory. IFN-γ is central to T cell-mediated antitumor activity. Signaling initiated by IL-12 and IFN-γ through STAT1 and STAT4 in Th cells induces T-bet, which, in turn, stimulates further IFN-γ production to promote a feed-forward loop. T-bet is required for optimal T cell IFN-γ generation, the differentiation of Th1 cells and the inhibition of suppressive Th subsets, including IL-4-induced Th2 cells, IL-6-IL/23-induced Th17 (IL-17-producing) cells, and TGF-β-induced regulatory T cells (Tregs) by acting on STAT6, STAT3, and forkhead box P3 (FoxP3), respectively [89]. Similarly, CD8 T cell release of IFN-γ through the direct interaction of pSTAT1 with the pro-survival *Bcl2a1* gene promoter impairs the suppressive function of MDSCs [90], while, in APCs, IFN-γ increases surface MHC class I and II expression along with other components of the antigen presentation pathway required for effective peptide recognition [54,91]. This extends to MHC class II induction on M1 macrophages, which can trigger IFN-γ release by Th1 cells to further activate macrophages that express abundant antitumor cytokines and factors, such as fas ligand (FASL) and nitric oxide (NO) [18], and directly phagocytose tumor cells [92].

Type II IFN also acts directly on tumor cells. Several studies have shown that IFN-γ halts tumor growth by inducing the tumor-intrinsic cell cycle inhibitor molecules p16 [50] and p21 [93]. Moreover, the upregulation of STAT1-dependent miR-29a/b in melanoma [94] has been evidenced to block tumor cell proliferation through the downregulation of cyclin-dependent kinase 6 [95]. An increase in apoptotic pathways through IFN-γ activation of STAT1-dependent caspase-1, -3, and -8 [96,97], along with cell death surface receptor FAS and its cognate ligand [98], has also been reported. 

The presence of type I IFN, often produced in large quantities by DCs, is required, for almost all aforementioned processes mediated by T cells [17]. IFN-α/β has been evidenced to promote the expansion and cytotoxic function in antigen-experienced CD8 T cells specifically via STAT4 and T-bet, beyond the role of type I IFN in DC recruitment and effective cross-priming for T cell activation [99]. Conversely, naïve cytotoxic T cells that lack IFNAR show impaired STAT1-dependent expansion and differentiation [100]. Likewise, *Irf7-/-* mice fail to launch durable CD8 T cell responses to toll-like receptor (TLR) bound peptides [39], due to the absence of type I IFN induction and pathway maintenance. Interestingly, the sensing of tumor-derived nucleic acids following cell death through TLRs that trigger JAK-STAT transduction to stimulate IRGs have been evidenced to act synergistically with T cell receptor engagement to enhance the T cell production of cytokines [101]. Moreover, it has been shown that IFN-α/β alone is sufficient for stimulating IFN-γ production by T lymphocytes [102]. Yet, despite the antitumor effects that are mediated by JAK-STAT signaling, there exists a flipside that drives the expansion and progression of cancer cells. 

## 4. Tumor Growth and Immune Evasion

The role of STAT3 in tumor development and progression is widely documented [103]. While the dysregulation of several microRNAs (miRs), such as let-7 [104] and miR-629 [105], might induce oncogenic transformation of epithelial cells through reciprocal STAT3 signaling, STAT3-driven tumor development largely results from loss of competent immune signaling and the induction of inflammatory responses in the TME [103]. Growth factors, such as vascular endothelial growth factor (VEGF) and TGF-β, in addition to a variety of cytokines, including IL-6, IL-17, IL-10, granulocyte-macrophage colony-stimulating factor (GM-CSF), and leukemia inhibitory factor (LIF), produced by both stromal and tumor cells, promote STAT3 activation. In a positive feedback loop, STAT3 subsequently transduces signals for all members of the IL-6 and IL-10 families [7], which leads to chronic amplification of pathways that support tumor growth.

In the TME, IL-6 trans-signaling promotes stable binding of GP130 and a soluble form of the IL-6 receptor (sIL-6R) to IL-6 [106]. Complex formation has been evidenced to drive JAK-STAT-dependent transendothelial migration of tumor cells [107], the recruitment of neutrophils and macrophages into the TME through endothelial cell secretion of monocyte chemoattractant protein-1 (MCP-1) [108], M2 polarization of macrophages, inhibition of DC activation, and Treg differentiation [109]. Myeloid cells chiefly regulate such mechanisms of sIL-6R-mediated immune suppression, along with IL-6-dependent inflammatory processes [103] in the TME have been linked to tumor progression and poor prognosis in several solid cancers, including gastric, breast [106], colorectal [110], ovarian [107], and lung [109]. 

While IL-6 is the most well-described activator of STAT3 signaling, the action IL-10 through STAT3 is somewhat more convoluted. Indeed, IL-10 remains controversial in the context of tumor formation, due to the often opposing downstream effects of α/β receptor binding and complex layers of regulation [111]. Highly expressed by Th17 cells, myeloid DCs, and TAMs [111] following the context-dependent activation of STAT3, auxiliary STATs, and extracellular signal-regulated kinase (ERK) pathways [111], the IL-10 family of cytokines have been reported to block cytotoxic T cell function through the inhibition of APCs [111] and promote Treg differentiation [112,113]. Moreover, in various cancers, high serum IL-10 is associated with shortened patient survival [114]. Conversely, IL-10 has been shown to limit inflammation, stimulate B cells, induce NK cell production of IFN-γ in concert with IL-18, and promote NK cell recognition of tumor cells, allegedly through the IL-10-mediated downregulation of tumor cell MHC [115]. Yet, while purportedly supporting early innate antitumor responses through the redirection of IL-10 signal transduction away from STAT3 and toward STAT1 by IFN-γ [116,117], the induction of secondary IL-10 responses subsequent to IFN-γ priming during tumorigenesis ultimately favors immune suppressive pathways, including those that are involved in Th2 differentiation, and diminishes the anti-inflammatory action of IL-10 [118]. 

In addition to STAT3, Th2 polarization is also reported to occur through STAT5 and STAT6 signal transduction. Early exploration of T helper cells demonstrated that JAK3-dependent STAT6 activation was required for IL-4-driven Th2 differentiation [119]. Later studies identified that STAT5, which was frequently overexpressed in breast and prostate cancer [1,120,121], could independently promote Treg development through the direct binding of the *FoxP3* promoter to stabilize expression [122]. STAT5 has also been implicated in IL-8 induction of tumor survival, chemotherapeutic resistance, and metastasis in breast cancer [121], with IL-8 expression in the TME known to drive TAM and neutrophil recruitment [123], further engendering an immune suppressive niche. 

The abundance and balance of cellular STATs is critical for cell-specific cytokine responses. For example, IFN-γ has been shown to enhance macrophage survival and proliferation in the absence of STAT1 via a poorly understood STAT1-independent mechanism that may result from alternative STAT3 activation, with associated loss of IL-12 production [124]—a cytokine critical to Th1 differentiation and cytotoxic function in T and NK cells. The overactivation of STAT5 has been shown to inhibit STAT1-mediated IFN-α responses and induction of IRGs in melanoma cells by increasing the expression of cytokine-inducible SH2 protein (CIS), which promotes ISRE suppression by blocking binding of STATs to JAK-associated IFNAR [125]. Similarly, STAT3 activation has been demonstrated to inhibit expression of STAT1, *Irf7*, and *Irf9* genes, effectively suppressing type I IFN signaling [126], which might contribute to the development of a poorly immunogenic TME. However, highlighting the complexity of heterogenous JAK-STAT activation in response to external stimuli that can give rise to a multitude of both pro- and antitumor immune responses, IFN-α/β has also been demonstrated to promote cytoplasmic complexation of STAT5 and oncoprotein CRKL, which induces select downstream IRGs through GAS element interactions [127]. This includes *Rap1*, which can render T cells unresponsive to antigen when constitutively expressed [128]. As such, the tight regulation of JAK-STAT pathways is crucial to the maintenance of homeostasis, which is achieved through negative regulation and extensive post-translational modifications of JAK-STAT members and intermediates.

## 5. STAT Signaling: Interactions and Inhibition

There is a surprising lack of redundancy in JAK-STAT signaling, given that several STATs may engage with the same DNA regulatory element (DRE), the same stimulus might activate multiple STATs, and heterogenous ligand interactions may act through the same STAT to induce differential downstream targets with disparate effects. As JAK-STAT pathways underpin such vast biological processes, including immune cell survival and regulation, strict homeostatic mechanisms work at numerous levels to ensure competent signaling is maintained [63]. This includes change-of-function signal transduction through the recruitment of auxiliary STATs, STAT competition, epigenetic modifications, and recruitment of proteins that inhibit JAK-STAT phosphorylation and DRE binding [2]. 

There are three main classes of negative regulators of JAK-STAT signaling. The production of suppressors of cytokine signaling (SOCS) proteins is stimulated upon STAT activation. SOCS proteins contain an SH2 domain that enables partner binding and a C-terminal SOCS box that directs the formation of an E3 ligase complex that facilitates proteolysis of SOCS-bound targets, including STATs, JAKs, and their associated receptors [129]. Several members of the SOCS family of proteins also contain kinase inhibitory regions (KIRs) adjacent to the SH2 domain that enable direct inhibition of JAK1, JAK2, and TYK2 enzymatic activity upstream of cytoplasmic STATs, which can also function to effectively block STAT docking sites [129]. In an added layer of JAK-STAT regulation, SOCS proteins may undergo hypermethylation, leading to transcriptional silencing, reported in various cancers, including liver and gastric carcinomas [130], in which the loss of SOCS3, and thus negative regulation of the IL-6/STAT3/NFκB axis, drives errant inflammation [131]. Similarly, loss of SOCS1, which is a critical mediator of T cell homeostasis, has been associated with the upregulation of known IRG PD-L1 and T cell inactivation resulting from uncontrolled IFN signaling [130]. Multiple miRNAs have also been shown to modulate SOCS expression [132]. In breast cancer, the suppression of SOCS1 by miR-155, which is required for effector T cell responses, resulted in enhanced JAK2-STAT3 signaling by more than three-fold, and it was linked to inflammation-driven tumor progression [133]. Likewise, hypermethylation of the SOCS3 promoter in response to loss of miR-122, which is heterogeneously expressed in liver tumors and associated with increased poor prognosis if downregulated, resulted in high STAT3 activation in hepatocellular carcinoma cells [134]. 

The rapid induction of SOCS upon STAT activation dictates the duration and intensity of a JAK-STAT response, as do the interactions between members of the PIAS (protein inhibitor of activated STAT) family and STAT dimers upon cytokine induction of JAK-STAT pathways. PIAS proteins, which possess E3 SUMO ligase activity, chiefly prevent STAT-mediated gene induction by physically blocking the DNA binding activity of translocated STATs in the nucleus [135], with the exception of PIASy and PIASx, which inhibit STAT signal transduction by recruitment of co-repressors, such as histone deacetylases (HDACs) [136]. PIAS1 is a potent inhibitor of STAT1-dependent type I and II IFN signaling, and it has been found to be upregulated in several malignancies, including metastatic prostate cancer [137,138]. Conversely, PIAS1 has also been demonstrated to block NFκB-induction of selective target genes in response to TNF-α [136,139]. This includes IL-1β, implicated in driving tumorigenesis and promoting TAM recruitment to the TME, thus highlighting the contextual and opposing functions of PIAS family members. In addition to SOCS proteins, studies have shown that the small and long non-coding RNA also regulates PIAS members [132]. Interaction between miRNA-18a and PIAS3 has been implicated in the development of gastric cancer through the overactivation of STAT3 when PIAS3 is silenced [132]. Similarly, in colorectal cancer the loss of lncRNA *Casc2* (Cancer susceptibility candidate 2), which normally functions to suppress errant STAT3 signaling by directing PIAS3 expression, has been associated with disease progression [140]. 

Phosphatases, such as SHP1, SHP2, DUSP2 [2], and CD45 [141], have also been implicated in the negative regulation of JAK-STAT signaling through their dephosphorylation of tyrosine residues on JAKs, STATs, and kinase-bound receptors, such as IFNAR [129], to facilitate rapid reactivation. Unsurprisingly, SHP2, which negatively regulates STAT1 and is heavily linked to T cell regulation, has been found to synergistically work with anti-PD1 to promote short-term antitumor immune responses when inhibited [142]. On the other hand, SHP1 has been found to act as a tumor suppressor in liver cancer by deactivating STAT3 and subsequent errant NFκB signaling [143]. 

Interestingly, direct members of the JAK-STAT pathway have been evidenced to both guide and execute epigenetic changes that lead to signal transduction alterations. In response to IFN stimulation, STAT1 and STAT2 have been shown to associate with HDAC1, while the chromatin remodeling factor, brahma-related gene 1, interacts with STAT2 to promote the recruitment of histone acetyltransferase (HAT) proteins p300 and CREB-binding protein (CBP) to mediate the transcription of IRGs [30]. Conversely, the anchoring of JAK2 in the nucleus has been linked to gene expression disruptions through direct phosphorylation of histones, such as H3Y41, which displaces the binding of heterochromatin protein 1α (HP1α) [144] to promote the loss of HP1α-mediated tumor and metastasis suppressive functions that occur through the regulation of genes that are associated with cell mitosis and adhesion [145,146]. Further tumor promoting effects have been evidenced through the nuclear localization of STAT5, which has been demonstrated to recruit the histone methyltransferase EZH2 (enhancer of zeste homolog 2) [147]. EZH2 has been linked to the progression on multiple solid cancers, including prostate [2,148], by silencing the genes that moderate proliferation and apoptosis. Notably, more recently, EZH2 has also been linked to Th1 inhibition in the TME [149]. Tumor-associated immune suppression has also been demonstrated through the upregulation of the fusion oncoprotein ETV6-NTRK3, implicated in breast cancer initiation, which simultaneously promotes STAT1 activation yet decreases STAT1 acetylation, subsequently inhibiting the acetylation of the NFκB subunit p65 to permit unchecked NFκB-driven inflammation [150].

Such diverse co-repressor and co-activator interactions broaden the transcriptional landscape of STAT signaling to give rise to a host of varied and distinct biological outcomes from the limited pool of JAK and STAT family members. The cross-regulation of STAT members explains why different STATs can have such opposing functions in response to the same stimulus. This is exampled by the induction of STAT1 and STAT3 by IFN-γ, whereby T cell differentiation is skewed toward Th1 by STAT1 and Th17 by STAT3, and that tumor cell proliferation might be restricted by STAT1 yet enhanced by STAT3—differential effects largely mediated by SOCS protein associations [89]. The complexity of regulation also explains why small changes in the accessibility, availability, and functionality of STATs and their auxiliary partners can have such profound effects on immune signaling and tumor progression.

## 6. Dysregulation of JAK-STAT Signaling in Cancer

The importance of JAK-STAT homeostasis and the consequences of perturbations have been widely explored in preclinical studies. JAK1 knockout mice demonstrate decreased cytokine responses, abnormal lymphocytic development, and die perinatally [63,151], while JAK2 knockout mice are embryonic lethal, as are STAT3 and STAT5 deficient mice [129]. Unsurprisingly, STAT1 deficient mice are unresponsive to both type I and II IFN signaling and, along with STAT2 knockout mice, are highly susceptible to infection indicative of impaired IFN-mediated immune development and maturation [30]. 

In certain cancers, JAK-STAT dysfunction has been frequently attributed to loss- or gain-of-function (LOF and GOF, respectively) mutations that may initiate and drive tumorigenesis [2]. Mutant JAKs can remain active and subsequently induce constitutive JAK-STAT signal transduction. Similarly, several mutations have been evidenced in STATs, particularly *Stat1*, *Stat3*, and *Stat5* [3,152]. Altered immune function is common to many of these mutations, including aberrant cytokine signaling and responsiveness, poor Th1 differentiation, IFN dysregulation, loss of NK cell cytotoxicity, abnormal myeloproliferation, and autoimmunity [63,152,153]. As such, many of the effects that LOF and GOF mutations in JAK-STAT pathway members direct in the cancer context are immune-associated. Somatic mutations in *Stat3* have been widely reported in multiple hematological malignancies [154]. Up to 72% of patients that are diagnosed with T-cell large granular lymphocyte (T-LGL) leukemia carry SH2-domain mutations in the *Stat3* gene, primarily Y640F GOF mutations [155], with alterations at this site being associated with chronic upregulation of *Irf7*, *Irf9*, *Ifngr2*, and *Bcl2l1* genes, suppression of the NFκB negative regulator *Birc3*, cytopenia, and errant myeloproliferation [3,156]. Similarly, *Stat5b* mutations have been linked to CD4^+^ T-LGL oncogenesis, with aberrant CD4^+^ T cell expansion being detected in up to 55% of patients [157]. In myeloproliferative cancer, the JAK2 V617F mutation is associated with significantly lower cumulative survival, whereby individuals harboring the mutation had a three-fold increased risk of early death as compared to individuals negative for JAK2 V617F [158]. While JAK-STAT mutations occur at lower frequencies in solid cancers [159], the JAK2 V617F mutation has also been observed in non-small cell lung cancer (NSCLC), with GOF mutations being linked to alterations in tumor cell PD-L1 [160]. Furthermore, in melanoma, LOF mutations in JAK1 and JAK2 have been linked to the loss of PD-L1 expression in the TME resulting from dampened tumor-inherent IFN signaling, which might contribute to poor patient response to checkpoint inhibitors [161]. Frameshift mutations at microsatellite repeats in JAK1 have been observed in prostate, urinary, and endometrial cancers, with the latter linked to loss of IRGs, including *Irf9*, and decreases in IFN-, IFN-γ, and complement Hallmark response genes, and reduced antigen presentation by immune cells in the TME [162]. Yet, while such definitive alterations have been observed in several cancers, many of the mechanisms that drive errant JAK-STAT signaling have not been described. Nevertheless, disrupted JAK-STAT signal transduction has been evidenced in many solid tumors. 

The aberrant expression of STAT3 and STAT5 in the TME is chiefly associated with tumor progression and spread. Increases in nuclear STAT5 have been linked to early recurrence and poor survival outcomes in prostate cancer, and overactivation of STAT3 that is associated with decreased survival and high risk of recurrence in renal cell carcinoma, glioblastoma, cervical cancer, colorectal cancer, and melanoma [1]. In addition to the amplification of oncogenes, such as *v-Src* (rouse sarcoma virus), *v-Abl* (Abelson murine leukemia virus), and *Egfr* (epidermal growth factor receptor), many roads lead to abnormal STAT3 phosphorylation. Persistent non-canonical STAT3 activation by the G-coupled protein receptor S1PR1 (sphingosine-1-phosphate receptor 1) in myeloid cells has been linked to the formation of a lung premetastatic niche in bladder cancer and melanoma models through the induction of factors that are associated with suppressive immune cell recruitment, invasion, angiogenesis, and tumor survival [163]. In prostate cancer bone metastases, which are largely poorly immunogenic, high tumor cell STAT3 phosphorylation, and IL-6R have been observed, as compared to lymph node and visceral metastases [164]. The increased expression of STAT3 and downstream targets, such as Survivin, has been demonstrated in multiple murine models of breast cancer, as well as patient tissues, and it has been linked to immune evasion during both tumorigenesis and metastasis [4,165]. In CD44^+^ CD24^−^ basal-like breast cancer cells—indicative of a stem cell-like phenotype—the overactivation of STAT3 correlated with low metastasis-free survival and the induction of pro-invasive chemokines, IL-6, and TGF-β signaling [166]. Yet, while the oncogenic effects of STAT3 have been well-established, activated STAT3 correlates with improved survival in some cancer subtypes and in combination with certain treatment regimens, [1], and recent studies have identified a tumor suppressor role for STAT3 [167]. This includes the attenuation of functional STAT3 by the STAT3β isoform, which is expressed in response to specific cytokine stimulation and by G-CSF secreted from myeloid cells during maturation and can abolish the activation of downstream targets and upregulate tumor cell apoptotic pathways that involve FAS—that is primarily induced by IFN-γ STAT1 activation [167]. Interestingly, in a somewhat opposing role, atypical STAT3 induction by IFN-γ during myeloid maturation by a subset of CD11b^+^ acute myeloid leukemia cells has been observed, which results in the upregulation of PD-L1 on tumor cells [168]. However, while this might contribute to immune evasion by promoting T cell exhaustion, it also makes them an attractive target for checkpoint inhibition. 

Where increased STAT3 activation is associated with tumor progression, it is often the loss of STAT1 and the associated pathway components that have been most widely explored in the context of cancer and immunity. In both melanoma and lung cancer models, impaired STAT1 phosphorylation in response to IFN-γ resulted in low MHC inducibility [169] and reduced IFN-γ sensitivity due to defective JAK signal transduction has been reported in human melanoma and lung carcinoma models [170]. Studies utilising STAT1 knockout mice have consistently reported the acceleration of tumor growth and defective IFN-γ-driven tumor cell killing by NK and T cells, indicating that both innate and adaptive antitumor responses are impaired when STAT1 is lost [6]. Interestingly, the expression of molecules, such as granzyme B (GZMB), perforin, and DAP10, which are regulated by STAT3 and mediate NK cell function and direct lytic activity, have been shown by Lee et. al. [26] to be comparable in *Stat1 -/-* mice as compared to wildtype counterparts. Despite this, the *Stat1 -/-* mice that were also T cell deficient were unable to reject NK cell-sensitive tumors, even in the presence of IL-12, which suggested that effective NK cell killing activity is still dependent on STAT1. In breast cancer, the loss of STAT1 in the mammary epithelium has been linked to *neu*-driven tumorigenesis [171] and spontaneous breast tumor development in BALB/C mice [172], with the latter being associated with loss of epithelial IRF1 and impaired T cell infiltration and killing. The *Brca1* (breast cancer type 1) gene, which is frequently mutated in breast and ovarian tumors, has emerged as a possible co-activator of type I IFN signaling [173]. Through complexation with nuclear STAT1 homodimers, BRCA1 has been proposed to directly modulate GAS binding through an IFN- γ—dependent mechanism that results in the upregulation of IRGs, such as *Irf7*. Thus, the loss of BRCA1 through germline mutation in malignant cells might aide in immune evasion and subsequent tumor outgrowth. 

While STAT1 is often discussed as promoting antitumor activity—the flipside of STAT3-driven protumor signaling—STAT1 induction has also been implicated in cancer progression. STAT1-dependent overexpression of IDO, which blocks T cell activation, has been observed in in several cancers [174] and is linked to high tumor cell PD-L1 in high-grade, triple-negative breast cancer (TNBC), while high U-STAT1 in the TME has been associated macrophage infiltration and poor outcomes in CD68-high patients in two mixed breast cancer subtype cohorts [175]. Similarly, the high tumor cell STAT1 has been linked to the recruitment of CD33^+^ myeloid cells to the TME, with increased infiltration correlating with progression from ductal carcinoma in situ to invasive carcinoma in women and murine models [176]. In numerous cancers, STAT1 overexpression has also been suggested to confer resistance to chemotherapy and radiation through the upregulation of IRGs that facilitate tumor cell survival, immune exhaustion through prolonged IFN-γ signaling, and errant growth [6,177]. Therapeutic resistance that is mediated by STAT3 in oncogene-driven cancers has also been observed [178]. Yet, the frequent and robust correlations of altered JAK-STAT pathways in cancer initiation and progression have made JAKs, STATs, and pathway intermediates attractive therapeutic targets.

## 7. Targeting STAT Signaling to Alter Tumor Progression 

The potential of JAK-STAT signaling intervention in pathogenesis has been known for many years. The extrinsic agonists and JAK-STAT pathway antagonists have been the most extensively trialed as ancillary STAT signaling modulators in an effort to block tumor progression and metastasis in a number of malignancies. Over the past decade, numerous agents that more directly target JAK-STAT elements, including JAKs, STATs, and SOCS family members, have also been explored to alter tumorigenesis, with more recent studies being focused on combination strategies with immune-targeted therapies. However, the versatility of upstream activators of JAK-STAT pathways and the pleiotropic nature of signal transduction makes them challenging to target.

### 7.1. TLR Induction of JAK-STAT Pathways 

The use of TLR inducers, including agents that mimic damage-associated molecular patterns (DAMPs), is undergoing a current resurgence and is gaining fast momentum in the field of cancer therapeutics. Yet, while numerous TLR agonists have progressed to clinical trial for the treatment of solid cancers, few have explored the direct impact on STAT signaling of such compounds in the TME [179,180]. Agents that stimulate TLR9 chiefly comprise of CpG oligodeoxynucleotides (ODNs), such as CpG7909 and SD-101 [181], have been shown to induce STAT1-dependent cytokine signaling in DCs [182], including IFN-α/β. Likewise, they have been linked to increased T cell fitness, MHC upregulation, and a reduction of immune suppressive populations in the TME [180,183], which likely result from indirect type I IFN stimulation via JAK-STAT signal transduction. While the phase III trial failure of CpG7909 to improve progression-free survival NSCLC [184] has hindered more extensive exploration of this particular compound beyond several phase I/II trials, including metastatic breast (NCT00043394) and prostate cancer (NCT00292045), for which the results have not been disclosed, several trials are ongoing with SD-101 in combination with checkpoint inhibition (NCT03831295; NCT03007732; NCT04050085 NCT02521870), with early reports of innate and adaptive immune cell infiltration and activation in the TME [185]. Early favorable results have also been documented in response to the TLR4 agonist, G100 [186], which also signals through STAT1 and synergizes with IFN-γ to induce a host of IRGs [187,188]. However, the TLR3 agonist Hiltonol™, which is a stabilized formulation of the potent IFN-inducer and JAK-STAT activator, poly I:C, is currently gaining the most traction, with over 44 clinical studies now underway in numerous solid cancers [179]. The jury is still out on the cellular impact and long-term clinical benefits of such compounds, particularly in regards to their use in combinatorial strategies and the promotion of autoimmunity, inflammation, and malignant cell survival in the TME [189]. Nevertheless, positive preclinical data [179,180,189,190,191] support the use of TLR-induction of JAK-STAT pathways to boost antitumor immunity, especially in metastatic settings, in which tumors are largely unreactive or immune-poor.

### 7.2. Cytokine Receptor Targeting 

Like TLR agonists, the use of recombinant cytokines, such as IL-12 [192], which is essential for STAT4-driven Th1 differentiation and the cytolytic function of effector T cells, and IL-15 [193], reported to increase NK cell activation and T cell expansion via STAT5 activation, have yielded successful outcomes in preclinical settings. Several phase I trials utilising the IL-15 superagonist ALT-803 as a monotherapy [193,194] or in conjunction with anti-PD1 [195] have recently commenced, with early reports of patient tolerance, immune activation, and antitumor activity disclosed in metastatic melanoma and NSCLC. Likewise, high-dose IL-2 has been under scrutiny in metastatic melanoma and renal cell carcinoma [196,197] for decades, with reports of disease stabilization and regression [198,199]. Yet, concomitant Treg induction has raised concerns regarding long-term application. In response, the IL-2 variants have been engineered that lack CD25 (IL-2Rα) binding and they can be fused to tumor-targeted antibodies, which are now entering clinical trial [199]. Likewise, numerous receptor-blocking antibodies have been approved to treat solid cancers. These include anti-IL-4 and Siltuximab, a monoclonal antibody targeting IL-6, which has been trialed in numerous solid cancers, yet, to date, has conferred no survival benefit [200]. In fact, Siltuximab was associated with increased tumor cell proliferation in prostate cancer, despite STAT3 dysregulation being frequently observed [132,200]. A repurposing of agents, such as mepolizumab and benralizumab, being used to block IL-5 signaling in asthma, is also on the rise [2,201]. However, the downfall of targeting or administering cytokines directly is that treatment often comes with significant toxicity [202]. Given the pivotal and context-dependent roles that many cytokines play in immune surveillance, tissue homeostasis, and cell survival, cytokine-targeting has also been associated with several side effects, including severe immune dysfunction, myocarditis, pruritus, and depression [202]. However, new delivery systems, including retroviral packaging and PEGylation of cytokines, may lower toxicity profiles. This has been evidenced through the approval of PEG-IFN-α2 as an adjuvant therapy in regional melanoma [202]. 

### 7.3. JAK Inhibition 

Several direct inhibitors of JAKs are also under investigation for use in multiple tumors. In widespread clinical trial for immune-related disorders, such as psoriasis, arthritis, and Crohn’s diseases, JAKinibs (JAK inhibitors), are now moving into the cancer space as combinatorial agents with other therapeutics, including checkpoint inhibitors [203]. The selective JAK1 inhibitor, Itacitinib, has been utilized in several phase Ib/II and III studies. One metastatic solid tumor phase Ib/II trial using Itacitinib with either *nab*-paclitaxel or gemcitabine (NCT01858883) reported partial responses with an overall response rate of 24% and acceptable safety profiles. Yet, the trial was terminated in early 2019 due to phase III results in two independent metastatic pancreatic cohorts while using JAK1/2 inhibitor ruxolitinib (NCT02117479; NCT02119663), which reported no survival benefit and significant toxicity, despite the success of the phase III MPACT trial (NCT00844649) investigating Itacitinib, also in combination with *nab*-paclitaxel and gemcitabine, in which the overall survival was significantly greater than patients treated with gemcitabine alone and in which 4% of patients demonstrated long-term remission above three years. Itacitinib is now in ongoing trials for metastatic solid tumors in combination with pembrolizumab (NCT02646748); NSCLC with Osimertinib (NCT02917993); and, BRAF-mutant melanoma with small-molecule MEK and BRAF inhibitors (NCT03272464), with outcomes yet to be reported. Modest survival benefit was reported in a phase II trial exploring Ruxolitinib in combination with Capecitabine in metastatic pancreatic cancer patients who failed gemcitabine [204], but the best responses were observed in patients with high C-reactive protein, which is a marker of inflammation. Ruxolitinib has also been trialed in metastatic TNBC [205]. Tumor cell decreases in JAK2 target genes (*Socs3*, *Egfr*) were reported, which indicated that target inhibition was achieved, along with decreases in activated STAT3, which was high at trial commencement in all patients. However, significant decreases in GZMB^+^ CD8^+^ T cells were observed in metastatic lesions compared to primary tumors, suggesting that JAK2 inhibition, which is critical in IFN responses and immune induction, particularly in a metastatic setting [206,207], might abrogate immune-mediated antitumor effects, as others have proposed. The trial was subsequently terminated due to progressive disease in all surviving enrolled patients. In fact, many JAKinibs that have shown promise in preclinical and early patient trials have failed to progress due to high toxicity and off-target immune-suppression, such as the JAK2 inhibitor, AZD1480 [152,208], which was shown to potently suppress both STAT1 and STAT3 signaling [209]. Such reports are increasingly salient, given the importance of retained type I IFN signaling in the TME to the efficacy of checkpoint inhibitors [190,210] that are often utilized in conjunction with JAKinibs, which JAK inhibition is likely to impede. As such, it might be unsurprising that no JAKinibs are currently FDA approved for cancer, despite their widespread usage in myelofibrosis. However, the continued development of new wave of JAKinibs with greater target specificity might lead to more promising future utility. 

### 7.4. Therapeutic Modulation of STATs 

Despite the fact that much preclinical work has been undertaken regarding the modulation of STATs and numerous promising STAT inhibitors developed or discovered, few have moved on to clinical trial [211]. Currently, the only STAT inhibitors undergoing clinical evaluation are three that target STAT3. OPB-31121, which is an SH2-binding STAT3 inhibitor that also interacts with STAT5, has shown antitumor activity in leukemia [212], and it has successfully undergone phase I trials in several solid tumors. Yet, to date, the mechanism of action and long-term consequences of STAT3 and STAT5 inhibition in the TME is poorly understood. The next-generation antisense oligonucleotide AZD9150, which targets the 3’ untranslated region of STAT3 to inhibit STAT3 protein, has demonstrated early clinical activity in lung cancer and lymphoma, with deceases in circulating tumor cells and MDSCs along with increases in CD8^+^ T cells reported in the latter [213]. It is also being trialed in lung, advanced pancreatic, and mismatch repair-deficient colorectal cancers in combination with the anti-PD-L1 checkpoint inhibitor, Durvalumab (NCT02983578); in advanced solid tumors in combination with Durvalumab and chemotherapy (NCT03421353); and, in treatment-refractory non-Hodgkin’s lymphoma in combination with acalabrutinib (NCT03527147), with no results reported at this time. Napabucasin, which inhibits STAT3-mediated transcription of target genes that regulate stemness, such as *Nanog*, is in phase III trial for patients with metastatic colorectal cancer in combination with the trichemotherapeutic, FOLFIRI (NCT03522649), and metastatic pancreatic adenocarcinoma in combination with *nab*-paclitaxel and gemcitabine (NCT02993731), along with several other phase I studies in other cancers, with positive patient outcomes being associated with safety and tolerance already reported in several solid tumor types [214,215,216]. Napabucasin has been demonstrated to sensitize tumor cells to checkpoint inhibition and it has been linked to high tumor infiltration of CD8^+^ T cells in mice bearing 4T1 mammary tumors [217], with similar results being reported in mesothelioma [218]. Notably, curcumin has demonstrated potent anti-STAT3 activity through STAT3 cysteine modification that prevents phosphorylation and it has been found to inhibit proliferation breast cancer [219] and esophageal squamous cell carcinoma [220], with the latter being associated with significant decreases in IL-6. However, while numerous studies using mice models have reported increases in TME infiltration by T cells, suppression of Tregs and MDSCs, and decreased NFκB signaling [221] in tumor bearing animals, curcumin has also been linked to the inhibition of DC activation [222,223]. Interestingly, ruxolitinib has been shown to target other kinases, including those that regulate DC recruitment (ROCK), which is suggested to contribute to the loss of DC activity observed in treated patients [152]. Therefore, it is possible that curcumin exerts the same effect, given it has also been shown to directly inhibit the activation of JAK2 [224]. 

The use of STAT1 activating agents, which comprise the recombinant cytokines previously discussed, along with immune stimulators, such as poly I:C, and select class-specific HDAC inhibitors (HDACi), have been consistently demonstrated to suppress STAT3 [124]. The cross-regulation of STAT1 and STAT3 and downstream target induction is suggested to occur via numerous mechanisms. These include, but are not limited to, the sequestering of cytoplasmic STAT3 in the presence of STAT1 through the formation of incompetent STAT1:STAT3 dimers, the suppression of STAT3 tyrosine phosphorylation, and concentration-dependent competitive DNA binding [89,225]. However, STAT1 modulation comes with its own set of problems. The upregulation of antiapoptotic and proinflammatory molecules, such as COX2 and iNOS, in response to STAT1 stimulation is frequently linked to tumorigenesis and cancer persistence. In particular, COX2 upregulation has been demonstrated to promote immune suppression, metastasis, and drug resistance in several cancer types [226,227,228], with all features being consistent with chronic STAT1 activation. Interestingly, the class I HDACi has been shown to inhibit iNOS, COX2, and arginase-1 in the MDSCs of tumor-bearing animals, in addition to enhancing the effectiveness of PD-1 inhibition to abrogate lung and renal tumor formation [229], which might be a product of STAT1 pathway inhibition. The prolonged exposure to both type I and II IFNs has been suggested to drive STAT1-mediated mechanisms of drug resistance. This is suggested to occur, in part, through the chronic induction of pro-survival cytokines, such as IL-6 and IL-8, in the TME and the selective elimination of tumor clones that retain sensitivity to IFN-dependent genotoxic or cytotoxic stress [177]. A multicancer screening identified 31 IRGs that were commonly upregulated in radioresistant patients, including *Cxcl10*, *Mcl1*, *Bst2*, *Ifitm*, *Usp18*, and *Stat1* [177,230], which formed the basis of a seven-gene IFN-related damage signature (IRDS). Further analysis in 295 early-stage breast cancer patients revealed that, of 243 that were further stratified for local-regional radiotherapeutic failure, up to 40% demonstrated high IRDS expression [231]. Likewise, STAT1 upregulation has been reported in resistance to doxorubicin, cisplatin, and docetaxel in ovarian and prostate cancer [6,177], while, in lung cancer, STAT1-dependent association with HDAC4 and the subsequent STAT1-driven upregulation of the multidrug resistance 1 (*Mrd1*) gene, has been linked to the failure of the topoisomerase II inhibitor, etoposide [232]. Interestingly, a translational switch has been identified, in which STAT1 promoted 5’ cap-independent induction of select genes that inhibit apoptosis (i.e., X-linked inhibitor of apoptosis, *xiap*) and enhance tumor cell viability [6], which might inform the future application of inhibitors through which to overcome resistance. Indeed, given the importance of STAT1 in competent antitumor immune surveillance, restoring JAK-STAT signaling balance through more targeted approaches may yield better outcomes than blanket and often leaky STAT1 modulation. 

## 8. Closing Statement

The increasing complexity and context-specificity of JAK-STAT signaling continues to be revealed through preclinical studies that have often yielded conflicting and surprising results. Such findings include the loss of effector cell function and increased metastasis in response to STAT3 inhibition [3], type I IFN-driven STAT3-dependent induction of cytotoxicity in tumor-infiltrating T cells to suppress tumor formation [233], and IFNAR1:STAT1-dependent Treg expansion and the production of IL-10 in the TME [234]. The cross-regulation and duplicity of JAK-STAT pathway mediators means that modulating a single target might not give rise to predicted phenotypical outcomes and may, in fact, generate undesired and often detrimental responses, as seen with the indiscriminate use of JAKinibs. While yet to be evaluated, it is currently unknown how such observations may extend to more recently proposed preclinical targets, such as SOCS family member CISH, shown to suppress both NK [235] and cytotoxic T [236] cell activity in tumor-bearing mice, which, when knocked out, can enhance antitumor immune function. Taken together, existing evidence suggests that JAK-STAT signaling members may ultimately better serve as diagnostic tools through which to stratify patients that may benefit from more targeted therapeutic approaches that modulate downstream targets, rather than upstream JAK-STAT pathway regulators. However, the selective and more tactical employment of direct JAK-STAT modulators might indeed overcome some of the challenges and adverse effects identified in prior investigations. Future exploration of JAK-STAT signaling in the context of cancer immunology will hopefully teach us how to better exploit such a critical informant of cancer evolution to enhance therapeutic strategies and predict those at the greatest risk of progression.
